# Empiric Transcatheter Arterial Embolization for Massive or Recurrent Gastrointestinal Bleeding: Ten-year Experience from a Single Tertiary Care Center

**DOI:** 10.7759/cureus.4228

**Published:** 2019-03-11

**Authors:** Azeemuddin Muhammad, Muhammad Awais, Raza Sayani, Muhammad Anwar Saeed, Saqib Qamar, Abdul Rehman, Noor U Baloch

**Affiliations:** 1 Radiology, The Aga Khan University, Karachi, PAK; 2 Radiology, Aga Khan University Hospital, Karachi, PAK; 3 Radiology, Rashid Hospital, Dubai, ARE; 4 Internal Medicine, Rutgers New Jersey Medical School, Newark, USA

**Keywords:** embolization, hemorrhage, gastrointestinal bleeding, empiric angioembolization, angiography

## Abstract

Purpose

In patients with massive or recurrent gastrointestinal bleeding (GIB) which is not amenable to endoscopic therapy, angiographic interventions are often employed. We report our ten-year experience of empiric transcatheter arterial embolization (TAE) for patients with massive or recurrent GIB.

Methods

All patients who had undergone empiric TAE at our hospital between March 2004 and June 2015 were identified using the institutional radiology information system. A retrospective chart review was performed using a structured pro forma. Technical success rate, 30-day clinical success rate, 30-day mortality rate, and rate of procedural complications were computed. Statistical analysis was performed using Statistical Package for Social Sciences (SPSS) version 20.

Results

A total of 32 patients had undergone empiric TAE for GIB during the study period. The median age of subjects was 56 years and two-thirds of them were male (68.7%). Gastroduodenal (n=24), ileocolic (n=3), left gastric (n=2), right gastroepiploic (n=1), and branches of superior and middle rectal arteries (n=1) were embolized using microcoils (n=25), polyvinyl alcohol particles (n=25), and gelatin sponge (n=3)--either alone or in combination. Technical and 30-day clinical success rates were 96.9% (31/32) and 71.9% (23/32), respectively. The 30-day mortality rate for our cohort was 21.9% (7/32). One patient developed re-bleeding at two days after the initial procedure and required repeat embolization. Coil migration (n=3) and access site hematoma (n=1) were the observed procedural complications.

Conclusion

Empiric TAE can be a useful treatment option for selected patients with massive or recurrent GIB that is not amenable to endoscopic therapy.

## Introduction

Gastrointestinal bleeding (GIB) accounts for more than 500,000 hospital admissions annually in the United States with a cumulative healthcare cost of nearly five billion dollars [1]. Massive GIB has been reported to have mortality rates as high as 24% and portends a worse prognosis [2]. Endoscopic therapy is considered the first-line treatment for patients with massive gastrointestinal hemorrhage [3-4]. However, this may not be possible in 8-25% of cases where endoscopic therapy may be unsuccessful [5], or may not be technically feasible [6]. In such cases, angiographic embolization or surgical ligation of bleeding vessels is the only available treatment option [7].

Transcatheter arterial embolization (TAE) is a commonly performed procedure and an estimated 1.7 million TAE procedures were performed in the United States in 2001 [8]. In patients with massive GIB, digital subtraction angiography (DSA) of the celiac, superior mesenteric and inferior mesenteric vessels can identify a source of bleeding in 30-47% of cases [9]. Demonstration of active contrast extravasation or pseudo-aneurysm formation is a tell-tale sign of active GIB and mandates TAE of the culprit vessel [10]. However, when DSA fails to demonstrate any overt signs of bleeding, the decision to perform TAE of a specific vessel becomes complicated. In many such cases, if the anatomic site of bleeding is known, 'empiric' TAE of the relevant vascular territory is performed [11].

The technical feasibility and clinical success of TAE in patients with angiographic evidence of active GIB has been well-established [12]. Moreover, the technical feasibility and clinical success of empiric TAE has also been demonstrated, mainly in patients with GIB due to duodenal ulcers [13-15]. However, in patients with massive or recurrent GIB due to other etiologies, empiric TAE is employed less commonly. Data on the safety of empiric TAE in cases of small intestinal or colonic bleeding is relatively scarce. In this retrospective study, we share our ten-year experience of performing empiric TAE for patients with massive or recurrent GIB due to a diverse array of etiologies.

## Materials and methods

A retrospective cross-sectional study was performed after obtaining an exemption from the institutional ethics review committee. We used the institutional radiology system to identify patients who had undergone empiric TAE for GIB at our institution between March 2004 and June 2015. Medical records for all these patients were retrieved and systematically reviewed using a structured pro forma. The data relating to demographics, co-morbidities, blood transfusions, laboratory investigations, endoscopies, radiologic studies, etiology of GIB, details of the TAE procedure, clinical outcome, and subsequent follow-up were specifically recorded. Personal identifiers or other identifiable information was not recorded. We excluded those patients who had less than 30 days of follow-up after the procedure. Etiology of GIB (if known) was based upon a final consensus diagnosis as recorded by the primary physician in the hospital chart.

Study site

Our hospital is a tertiary care center located in a densely populated city of a low-to-middle income country. The hospital has a dedicated vascular and interventional radiology suite with state-of-the-art facilities. This suite remains operational from 8 AM to 5 PM during which elective procedures are performed. Emergency procedures during daytime are performed rapidly by temporarily suspending elective procedures. For the afterhours, a dedicated on-call interventional radiology team consisting of technicians and a consultant interventional radiologist is on standby. When consulted by treating physicians, the on-call team is mobilized and the interventional radiology suite is activated for emergency procedures. Interventional procedures are performed by three different consultant interventional radiologists, all of whom have completed post-graduate training in radiology and dedicated fellowships in interventional radiology. Also, all of them have more than five years of experience in performing interventional radiologic procedures.

Protocol for management of GIB

Emergency physicians, gastroenterologists, and general surgeons consult the on-call vascular and interventional radiology team for many patients with GIB. All patients undergoing TAE for GIB at our institution have either massive or recurrent GIB where endoscopy and/or surgery have failed, or are deemed unfeasible. In most patients with massive GIB, computed tomography (CT) with GIB protocol is obtained prior to DSA in an attempt to localize the source of bleeding. The decision to perform DSA is based on a consensus between the on-call interventional radiologist and the primary treating physician. In patients who undergo DSA, TAE is always attempted if angiographic evidence of active bleeding (active contrast extravasation, pseudo-aneurysm formation, presence of arteriovenous fistulae or abnormal vascular blush) is evident. In cases when angiographic findings of active bleeding are absent, the decision to perform empiric TAE or not is taken by the performing interventional radiologist. In most patients with recurrent GIB, the anatomic source of bleeding is evident based on results of previous endoscopies, radiologic studies and/or operative findings, and empiric TAE is attempted in such cases. However, in some patients with massive GIB, the anatomic source of bleeding may not be known with certainty; in such cases, empiric TAE is not performed if the interventional radiologist believes that the anatomic source of bleeding is not known with certainty.

Interventional radiology procedures

For all patients included in the study, DSA procedures were performed in the interventional radiology suite using a flat panel monoplane DSA machine (Angiostar®, Siemens Medical Systems; Erlangen, Germany). The right or left groin was punctured to gain access to the common femoral artery using a 4-Fr or 5-Fr vascular access sheath (Cordis; Miami, FL, USA). Selective cannulation of the celiac, superior mesenteric and inferior mesenteric arteries was then performed. Diagnostic angiographies were typically carried out using 4-Fr cobra (C1) cordis, renal double curve (RDC), cordis or Simmons (SIM1) cordis catheters. TAE was performed by a super-selective cannulation of suspected target vessels using a microcatheter (Progreat, Terumo; Tokyo, Japan) followed by deployment of vascular occlusive microcoils (Balt Extrusion; Montmorency, France), polyvinyl alcohol (PVA) particles (Boston scientific; Marlborough, MA, USA), and/or gelatin sponges (Gelfoam, Pharmacia & Upjohn; Kalamazoo, MI, USA). At our interventional radiology suite, N-butyl cyanoacrylate (NBCA) glue (Histoacryl, B. Braun; Melsungen, Germany) is also available; however, it was not utilized by any consultant interventional radiologist for performing empiric TAE among the subjects included in this study. Following empiric TAE, repeat angiograms were performed to determine the adequacy of TAE and to assess collateral vessels.

Operational definitions and statistical analysis

The technical success of TAE was defined as complete angiographic occlusion of the suspected culprit vessel. The technical success rate was calculated as the ratio of the number of technically successful empiric TAE procedures to the total number of procedures where a decision to perform empiric TAE was made by the performing interventional radiologist. Clinical success was determined by a combination of technical success and no evidence of on-going GIB (as suggested by clinical, laboratory and endoscopic findings) within the first 30 days after the procedure. For the purpose of this study, the patients who died within the first 30 days of the empiric TAE procedure were considered to have clinical failure. To calculate the 30-day clinical success rate, we divided the number of patients with clinically successful empiric TAE procedures to the total number of procedures where a decision to perform empiric TAE was made. The 30-day mortality rate for our study cohort was computed as the ratio of the number of patients alive at 30 days after the empiric TAE procedure to the total number of patients included in this study. Statistical package for social sciences (SPSS) version 20.0 was used for performing statistical analysis. Frequencies were calculated for qualitative variables, while the median (inter-quartile range [IQR]) were computed for quantitative variables. We did not use any statistical tests as no comparisons were performed in this descriptive study.

## Results

A total of 32 subjects were included in this study with a predominance of men (n=22, 68.7%). The median age of study subjects was 56 (IQR: 32-78) years. Duodenal ulcer (n=16, 50%) was the most common etiology of GIB followed by malignancy (n=7, 21.9%), undetermined etiology (n=4, 12.5%) and gastric ulcer (n=2, 6.2%). Most patients (n=29, 90.6%) had one or more medical comorbidities with the most common being hypertension (n=25, 78.1%) and diabetes mellitus (n=18, 56.2%). Laboratory evidence of abnormal hemostasis was evident in 11 (34.4%) cases. The median nadir hemoglobin for our patients was 6.8g/dl (IQR: 5.2-7.1). Packed red blood cells were transfused in all patients prior to TAE with a median number of 12 (IQR: 6-18) units transfused per patient. These demographic characteristics are further summarized in Table 1.

**Table 1:** Characteristics of patients included in our study (n = 32) *IQR* = interquartile range; *RBC* = red blood cell.

Characteristics	Results (n [%])
Sex	
Male	22 (68.7%)
Female	10 (31.2%)
Age (median [IQR])	56 (32–78) years
Etiology of gastrointestinal hemorrhage	
Duodenal ulcer	16 (50.0%)
Malignancy	7 (21.9%)
Undetermined	4 (12.5%)
Gastric ulcer	2 (6.2%)
Pancreatitis	1 (3.1%)
Glanzmann thrombasthenia	1 (3.1%)
Aorto-enteric fistula	1 (3.1%)
Medical co-morbidities	
Hypertension	25 (78.1%)
Diabetes mellitus	18 (56.2%)
Malignancy	10 (31.2%)
Ischemic heart disease	7 (21.9%)
Cerebrovascular disease	4 (12.5%)
Liver cirrhosis	3 (9.4%)
Others	3 (9.4%)
Nadir hemoglobin (median [IQR])	6.8g/dl (5.2–7.1)
Number of packed RBC transfused (median [IQR])	12 (IQR: 6–18) units per patient

The anatomic sites of GIB were duodenum, stomach, cecum, and rectum in 25 (78.1%), 3 (9.4%), 3 (9.4%), and 1 (3.1%) cases, respectively. Empiric TAE was technically successful in 31/32 patients affording a technical success rate of 96.9%. In the particular case where TAE was technically unsuccessful, the unusual anatomy of the gastroduodenal artery was noted on DSA. The gastroduodenal artery was filling retrogradely from the superior mesenteric artery with subsequent retrograde filling of the hepatic and gastroepiploic arteries. Of the 31 patients that had empiric TAE, seven patients died within the first 30 days giving a 30-day mortality rate of 21.9%. The most common causes of death included respiratory failure, myocardial infarction, and septic shock. The cause of death was unrelated to the procedure in all cases. One patient developed re-bleeding two days after undergoing TAE of the gastroduodenal artery. This patient had Glanzmann thrombasthenia and, during the initial TAE procedure, a microcoil had migrated inadvertently from gastroduodenal artery into the hepatic artery. Upon performing repeat DSA, active contrast extravasation was noted from the gastroduodenal artery, which was filling retrogradely through collateral vessels. Repeat embolization of the culprit vessels was performed successfully with two microcoils. However, the patient developed a groin hematoma from the repeat procedure. Subsequently, he had no evidence of GIB and remained clinically stable afterwards. Re-bleeding was not noted in any other patient. The 30-day clinical success rate was calculated to be 71.9% (23/32). The procedural complications that occurred in our study participants were coil migration and groin hematoma, which occurred in 3 (9.4%) and 1 (3.1%) cases respectively. In all three cases of coil migration, patients had extensive aorto-iliac atherosclerosis, which rendered the manipulation of angiographic catheters extremely difficult. In all such cases, microcoils inadvertently migrated from the gastroduodenal artery onto the right hepatic artery. However, in such cases, there was no significant restriction of blood flow to the liver and none of these subjects had any clinical evidence of abnormal hepatic function. The only patient who developed a groin hematoma had a history of Glanzmann thrombasthenia and did well with conservative management. Asymptomatic duodenal stenosis was discovered in three cases on post-TAE upper endoscopy, which did not require any specific treatment. Other TAE-related complications, such as arterial dissection, bowel infarction, acute renal failure or contrast reactions, were not observed in our study cohort. The median duration of follow-up for our patients was 63 (IQR: 33-102) days.

**Figure 1:**
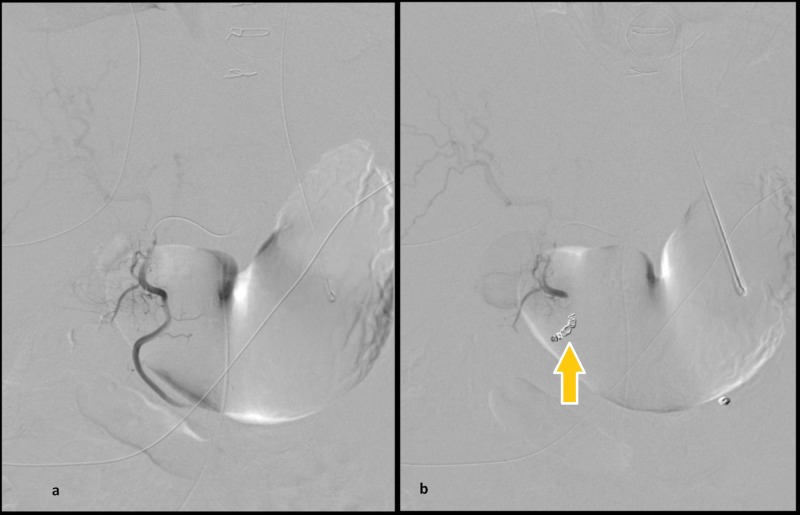
Empiric transcatheter arterial embolization of the gastroduodenal artery (a) Pre-embolization angiogram fails to reveal active contrast extravasation from the gastroduodenal artery. (b) Post-embolization angiogram shows multiple microcoils (arrow) in the gastroduodenal artery with no flow of contrast distally.

**Figure 2:**
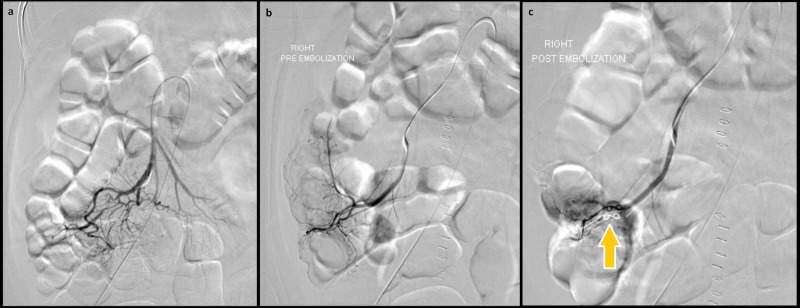
Empiric transcatheter arterial embolization of the ileocolic artery (a), (b) Pre-embolization angiograms fail to reveal any active contrast extravasation from the ileocolic artery. (c) Post-embolization angiogram shows a microcoil (arrow) in the ileocolic artery with significantly reduced vascularity.

**Figure 3:**
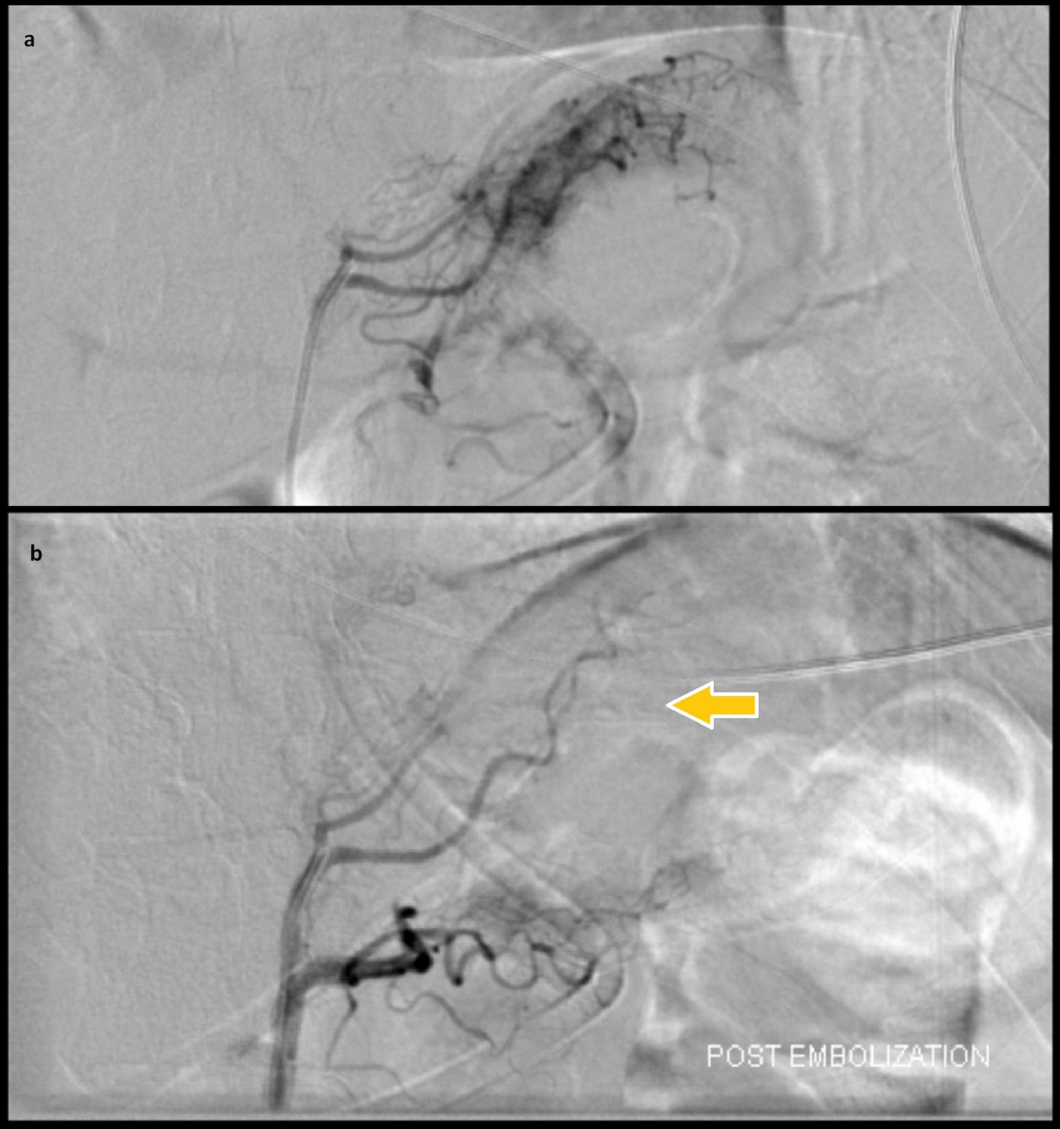
Empiric transcatheter arterial embolization of the left gastric artery (a) Pre-embolization selective left gastric angiogram shows no angiographic evidence of active bleeding. (b) Post-embolization angiogram shows significantly reduced vascularity in the region of gastric fundus (arrow) near the gastro-esophageal junction. Arterial embolization in this case was performed with polyvinyl alcohol particles (355–500 μm).

Among patients who underwent TAE, the most commonly embolized vessels were the gastroduodenal artery (n=24, 75%; Figure 1), the ileocolic artery (n=3, 9.4%; Figure 2), and the left gastric artery (n=2, 6.2%; Figure 3) as given in Table 2. A combination of microcoils and PVA particles (n=19, 59.4%) was used in most cases, followed by PVA particles alone (n=5, 15.6%) and microcoils alone (n=3, 9.4%). Microcoils and gelatin sponge were used in two (6.2%) cases, while in one (3.1%) case, a combination of microcoils, PVA particles, and gelatin sponge was utilized. NBCA glue was not used for empiric TAE in any case. Size of PVA particles used in our study ranged from 355 μm to 500 μm.

**Table 2:** Results of transcatheter arterial embolization performed in study subjects (n = 32) *PVA* = polyvinyl alcohol.

N	Anatomic sites	Etiologies	Technical success	Vessels embolized	Agents employed	Clinical outcome
24	Duodenum	Duodenal ulcer (n=16), malignancy (n=6), Glanzmann thrombasthenia (n=1), aorto-enteric fistula (n=1), undetermined (n=1)	23/24	Gastroduodenal artery (n=23)	PVA particles (n=19), microcoils (n=22), gelatin sponge (n=2)	Success (n=16), re-bleeding (n=1), died (n=7)
3	Stomach	Gastric ulcer (n=2), pancreatitis (n=1)	3/3	Left gastric artery (n=2), gastroduodenal and right gastro-epiploic arteries (n=1)	PVA particles (n=3), microcoils (n=1)	Success (n=3)
3	Cecum	Undetermined (n=3)	3/3	Ileocolic artery (n=3)	PVA particles (n=2), microcoils (n=1)	Success (n=3)
1	Rectum	Malignancy (n=1)	1/1	Branches of superior and middle rectal arteries (n=1)	Microcoils + PVA particles + gelatin sponge (n=1)	Success (n=1)

## Discussion

Our study demonstrates that empiric TAE is a technically feasible procedure in patients with a known anatomic site of GIB where endoscopic or surgical therapy has failed, or is deemed unfeasible. The clinical success rate (71.7%) that we reported in our study is comparable to that reported by previous studies [5,7,10-15]. Aina et al. reported a clinical success rate of 76% in a cohort of 75 patients who underwent TAE for upper GIB [11]. Likewise, Ichiro and co-workers found that empiric TAE for bleeding duodenal ulcers had a clinical success rate of 83% [14]. Furthermore, in another study, Dixon and colleagues showed that the clinical success rate for empiric TAE (80%) in patients with upper GIB was comparable to that for TAE (85%) performed in patients with angiographic evidence of upper GIB [16].

In patients with recurrent or massive GIB, angiographic findings of hemorrhage (active contrast extravasation, pseudo-aneurysm formation or arterial spasm) may not always be evident [2]. In cases where angiographic evidence of GIB is absent, 'empiric' or 'blind' TAE may be performed in the anatomic site of GIB [7]. Although such a procedure is termed 'empiric' or 'blind', the anatomic site of GIB is usually certain based on radiologic, endoscopic, and/or operative findings [5]. Such a technique of performing TAE in the absence of angiographic evidence of active bleeding is also employed for various other purposes in different case scenarios. For instance, TAE is often employed pre-operatively for highly vascular tumors to reduce their vascularity and ameliorate the severity of intra-operative bleeding [17].

The absence of angiographic evidence of bleeding does not preclude recent, or even active, GIB. In many patients, GIB is intermittent and may not be evident at the time of DSA [18]. Moreover, in patients with active GIB, systemic hypotension can mask frank contrast extravasation at the site of bleeding. Animal-based models of conventional angiography suggest that catheter-based DSA cannot detect bleeding if it occurs at a rate of less than 0.5 mL per minute [19]. Additionally, vessels may go into vasospasm and this may be another reason for the absence of angiographic contrast extravasation [20]. Due to the aforementioned reasons, empiric TAE can establish hemostasis and effectively control hemorrhage in patients with massive or recurrent GIB provided the anatomic site of bleeding is certain and the target vessels are embolized completely [11].

The 30-day clinical success rate in our study was comparable to other studies [5,7,10-15]. However, there is considerable discrepancy amongst published studies regarding the definition of “clinical success rate” [13]. In our study, we excluded patients who died from any cause and considered them as failures for the purpose of this calculation. The reason for doing this was to increase the power of the study as attributing death to GIB or the procedure is extremely subjective. For instance, myocardial infarction, respiratory failure and/or septic shock can frequently occur as sequelae of prolonged, uncontrolled hemorrhage. Given the subjectivity and bias inherent in such a determination, we decided to deem all patients, who died of any cause within the first 30 days after empiric TAE, as clinical failures. However, not all investigators concur with this method of calculating clinical success rate [10-15].

Efficacy of different embolic agents used for TAE could not be assessed in this small retrospective study. However, NBCA glue was not used in any case by our interventional radiologists. Traditionally, NBCA glue has been avoided for performing TAE in patients with GIB due to fear of spillage of NBCA glue into nearby vessels [7]. However, in a recent systematic review, Kim and colleagues analyzed 440 cases from 15 different studies and reported that NBCA glue is a safe and effective agent for embolization of vessels in patients with GIB [21]. In the present study, under-utilization of NBCA glue was most likely a combined consequence of radiologists’ personal preferences and a lack of experience in using NBCA glue for such cases.

The most common complication of TAE is re-bleeding, which has been previously reported to occur in approximately 11-24% of cases [11,14,16]. The rate of re-bleeding observed in our study was much lower than that reported in the literature. This may be attributable to meticulous procedural technique and experience of individual radiologists in performing TAE and employing the use of multiple embolic agents to secure hemostasis. However, a substantial proportion of patients in our study cohort died within the first 30 days (21.9%), which may have led to an underestimation of re-bleeding events. Having said that, the 30-day mortality rate of our study cohort (21.9%) is in line with observations of previous studies; the mortality of massive GIB is estimated to be as high as 24% [2]. In the only patient who developed re-bleeding in our study, TAE procedure was complicated by coil migration and re-bleeding occurred two days after the procedure. This observation is consistent with published literature which suggests that early re-bleeding after TAE is most likely a consequence of poor technique [11]. From a theoretical perspective, culprit vessels go into vasospasm at the time of TAE, which would provide a false impression of hemostasis. Later, when such vessels dilate and re-open, they provide a source of collateral flow and result in re-bleeding [22]. Based on this premise, the use of provocative angiography has been explored by some researchers [23], although its feasibility and safety remains to be established. Provocative angiography is not employed at our institution due to safety concerns that fibrinolytics can potentially worsen GIB and vasodilators may precipitate lethal hypotension in critically ill patients with massive GIB [14].

A number of other complications have been reported in patients undergoing TAE for GIB [7]. Arterial dissection, bowel infarction, and anaphylaxis are rare complications, which were not observed in our study cohort. Access site-related complications, such as hematoma and pseudo-aneurysm formation, have been reported to occur in a small subset of patients [11]. In our study, only one patient developed groin hematoma and did well with conservative management; this patient had a history of Glanzmann thrombasthenia. Hepatic infarction and fulminant hepatic failure have been reported to occur in some patients undergoing TAE for GIB [7,11]. In our study, inadvertent coil migration occurred in three cases and, in all these cases, microcoils migrated from the gastroduodenal artery into the hepatic artery. However, evidence of hepatic failure or dysfunction was not observed in any case. Duodenal ischemia is a known complication of TAE of the gastroduodenal artery and may be detected in later stages as duodenal stenosis [7,11,15]. In our study, symptomatic duodenal ischemia did not occur in any patient, but, asymptomatic duodenal stenosis was later detected in only three cases. Moreover, among patients who underwent ileocolic artery and rectal artery TAE, intestinal infarction or ischemic procto-colitis did not occur in any patient.

Ileocolic artery TAE is a less commonly performed procedure and limited literature is available to support its technical feasibility and safety [10,24]. In the three patients who underwent this procedure in our study, the anatomic site of bleeding was confirmed by CT with GIB protocol. As mentioned previously, CT with GIB protocol is performed for most patients with massive GIB at our institution prior to diagnostic angiography. In a previous study published in 2016, we showed that CT with GIB is superior to RBC scintigraphy in terms of detecting GIB and localizing an anatomic site [25]. Some investigators do not favor this approach due to the fear of increasing the incidence of acute kidney injury and losing precious time in performing the CT scan [14]. However, modern multi-row CT scanners with automated bolus tracking techniques can perform such scans within a span of five minutes. Furthermore, in a study published in 2015, Jacovides and co-workers showed that use of such a diagnostic algorithm improves localization and yield of diagnostic angiography without increasing the incidence of acute kidney injury among such subjects [26].

This study does come with a number of caveats. First of all, our study was a retrospective charts review and improper documentation might have led to an underestimation of certain complications. However, our hospital is accredited by the Joint Commission International and special emphasis is placed on proper documentation and accurate charting. Secondly, we only reviewed the charts of patients who underwent empiric TAE for GIB in this study and did not have a control group. But, the results observed in our study were comparable to those reported by previous studies, which suggests that TAE is a feasible procedure. Additionally, our study had a small sample size, which precluded the determination of predictors of re-bleeding or mortality. Lastly, our study was from a single tertiary care center in a developing country; therefore, the rate of complications observed in our study may not be generalizable to centers of other lower-income countries where experienced radiologists may not always be available. Despite these limitations, our study did provide some evidence in support of the feasibility and safety of empiric TAE as a therapeutic option for patients with GIB.

## Conclusions

Empiric TAE is a technically feasible and safe therapeutic option for patients with recurrent or massive GIB from a known anatomic source where endoscopic therapy has failed, or is deemed unfeasible.

## References

[1] Peery AF, Dellon ES, Lund J (2012). Burden of gastrointestinal disease in the United States: 2012 update. Gastroenterology.

[2] Wu LM, Xu JR, Yin Y, Qu XH (2010). Usefulness of CT angiography in diagnosing acute gastrointestinal bleeding: a meta-analysis. World J Gastroenterol.

[3] Gralnek IM, Dumonceau JM, Kuipers EJ (2015). Diagnosis and management of nonvariceal upper gastrointestinal hemorrhage: European Society of Gastrointestinal Endoscopy (ESGE) guideline. Endoscopy.

[4] Strate LL, Gralnek IM (2016). ACG clinical guideline: management of patients with acute lower gastrointestinal bleeding. Am J Gastroenterol.

[5] Eriksson LG, Ljungdahl M, Sundbom M, Nyman R (2008). Transcatheter arterial embolization versus surgery in the treatment of upper gastrointestinal bleeding after therapeutic endoscopy failure. J Vasc Interv Radiol.

[6] Seif HM, Moustafa EF (2010). Prophylactic embolisation of the left gastric artery in cases with massive upper gastrointestinal haemorrhage with normal angiographic findings. Arab J Gastroenterol.

[7] Defreyne L, Vanlangenhove P, de Vos M (2001). Embolization as a first approach with endoscopically unmanageable acute nonvariceal gastrointestinal hemorrhage. Radiology.

[8] Bhargavan M, Sunshine JH (2005). Utilization of radiology services in the United States: levels and trends in modalities, regions, and populations. Radiology.

[9] Barnert J, Messmann H (2009). Diagnosis and management of lower gastrointestinal bleeding. Nat Rev Gastroenterol Hepatol.

[10] Bandi R, Shetty PC, Sharma RP, Burke TH, Burke MW, Kastan D (2001). Superselective arterial embolization for the treatment of lower gastrointestinal hemorrhage. J Vasc Interv Radiol.

[11] Aina R, Oliva VL, Therasse É, Perreault P, Bui BT, Dufresne MP, Soulez G (2001). Arterial embolotherapy for upper gastrointestinal hemorrhage: outcome assessment. J Vasc Interv Radiol.

[12] Tan KK, Wong D, Sim R (2008). Superselective embolization for lower gastrointestinal hemorrhage: an institutional review over 7 years. World J Surg.

[13] Arrayeh E, Fidelman N, Gordon RL, LaBerge JM, Kerlan Jr RK, Klimov A, Bloom AI (2012). Transcatheter arterial embolization for upper gastrointestinal nonvariceal hemorrhage: is empiric embolization warranted?. Cardiovasc Intervent Radiol.

[14] Ichiro I, Shushi H, Akihiko I, Yasuhiko I, Yasuyuki Y (2011). Empiric transcatheter arterial embolization for massive bleeding from duodenal ulcers: efficacy and complications. J Vasc Interv Radiol.

[15] Poultsides GA, Kim CJ, Orlando III R, Peros G, Hallisey MJ, Vignati PV (2008). Angiographic embolization for gastroduodenal hemorrhage: safety, efficacy, and predictors of outcome. Arch Surg.

[16] Dixon S, Chan V, Shrivastava V, Anthony S, Uberoi R, Bratby M (2013). Is there a role for empiric gastroduodenal artery embolization in the management of patients with active upper GI hemorrhage?. Cardiovasc Intervent Radiol.

[17] Salai M, Garniek A, Rubinstein Z, Segal A, Morag B (1999). Preoperative angiography and embolization of large pelvic tumors. J Surg Oncol.

[18] Fiorito JJ, Brandt LJ, Kozicky O, Grosman IM, Sprayragen S (1989). The diagnostic yield of superior mesenteric angiography: correlation with the pattern of gastrointestinal bleeding. Am J Gastroenterol.

[19] Baum S (1982). Angiography and the gastrointestinal bleeder. Radiology.

[20] Kim BSM, Li BT, Engel A, Samra JS, Clarke S, Norton ID, Li AE (2014). Diagnosis of gastrointestinal bleeding: A practical guide for clinicians. World J Gastrointest Pathophysiol.

[21] Kim PH, Tsauo J, Shin JH, Yun SC (2017). Transcatheter arterial embolization of gastrointestinal bleeding with N-butyl cyanoacrylate: A systematic review and meta-analysis of safety and efficacy. J Vasc Interv Radiol.

[22] Shah AA, Rehman A, Haider AH (2015). Angiographic embolization for major trauma in a low-middle income healthcare setting—A retrospective review. Int J Surg.

[23] Kim CY, Suhocki PV, Miller Jr MJ, Khan M, Janus G, Smith TP (2010). Provocative mesenteric angiography for lower gastrointestinal hemorrhage: Results from a single-institution study. J Vasc Interv Radiol.

[24] Evangelista PT, Hallisey MJ (2000). Transcatheter embolization for acute lower gastrointestinal hemorrhage. J Vasc Interv Radiol.

[25] Awais M, Haq TU, Rehman A, Zaman MU, Haider Z, Khattak YJ, Baloch NU (2016). Accuracy of 99mTechnetium-labeled RBC scintigraphy and MDCT with gastrointestinal bleed protocol for detection and localization of source of acute lower gastrointestinal bleeding. J Clin Gastroenterol.

[26] Jacovides CL, Nadolski G, Allen SR (2015). Arteriography for lower gastrointestinal hemorrhage: role of preceding abdominal computed tomographic angiogram in diagnosis and localization. JAMA Surg.

